# Characterization of Electrical Heating Textile Coated by Graphene Nanoplatelets/PVDF-HFP Composite with Various High Graphene Nanoplatelet Contents

**DOI:** 10.3390/polym11050928

**Published:** 2019-05-27

**Authors:** Hyelim Kim, Sunhee Lee

**Affiliations:** 1 Research Institute of Convergence Design, Dong-A University, Busan 49315, Korea; hyelim1221@gmail.com; 2 Department of Fashion Design, Dong-A University, Busan 49315, Korea

**Keywords:** graphene nanoplatelet, high-content, horseshoe pattern, silver coated conductive yarn, electrical heating textile, electrical heating property

## Abstract

We prepared a horseshoe-pattern type electrical heating textile that was coated with high graphene nanoplatelet (GNP) content (32 wt% to 64 wt%) of graphene nanoplatelet/poly(vinylidene fluoride-*co*-hexafluoropropylene) (PVDF-HFP) composite. Silver-coated conductive yarn is used as electrode in the sample to improve its flexibility and applicability as wearable textile. These graphene nanoplatelet/PVDF-HFP coated samples with various high-contents of graphene were characterized using scanning electron microscopy (SEM), thermogravimetric analysis (TGA), differential scanning calorimetry (DSC), X-ray diffraction (XRD), sheet resistance analysis, and electrical heating performance analysis. Graphene nanoplatelet/PVDF-HFP coated cotton fabric improved the crystallinity and thermal stability with increasing thw high-content of GNP. With an increasing of the high-content of graphene nanoplatelet in the PVDF-HFP composite solution, the sheet resistance of samples tended to gradually decrease. That of, 64 wt% graphene nanoplatelet/PVDF-HFP composite coated sample (64 GR/cotton) was 44 Ω/sq. The electrical heating performance of graphene nanoplatelet/PVDF-HFP composite coated cotton fabric was improved with increasing the high-content of graphene nanoplatelet. When 5 V was applied to 64 GR/cotton, its surface temperature has been indicated to be about 48 °C and it could be used at a low voltage (<10 V). Thus, a horseshoe-pattern type electrical heating textile that is coated by high content of graphene nanoplatelet/PVDF-HFP composite solution sewn with silver-coated conductive yarn is expected to be applied to glove, shoes, jacket, and so on to improve its wearability and applicability.

## 1. Introduction

Electronic textiles (E-textiles) are emerging and gradually being integrated into our homes and daily life due to their potential applications in conductive fabrics, flexible sensors, and wearable heaters [1,2]. Recently, carbon-based nanomaterials, such as carbon nanotubes (CNT), carbon nanofiber (CNF), and graphene are used to satisfy the need for mechanically flexible, highly conductive, wearable and lightweight material due to their unique mechanical property and electrical and thermal conductivities [3,4]. They are used as conductive fillers in the polymer-based composites. Among the carbon-based nanomaterials, the graphene family can be classified as single layer graphene, few layer graphene (2–10 layers), and graphite nano- and micro-platelets by the morphological characteristics [5]. Graphene nanoplatelets (also known as graphite nanoplatelets, GNPs, or GPs) is one of graphene family. It can be used to prepare a stacked two-dimensional graphene sheet ideal nano-filler to enhance the polymer matrix due to its good aspect ratio, unique two-dimensional structure, and low manufacturing cost [6,7]. Using the unique property of GNPs, previous studies have prepared films and conductive textiles with graphene/polymer-based composite with various graphene contents [8,9,10,11,12,13,14]. Hu et al. [10] have prepared the graphene/PVDF films with a high-content of graphene, ranging from 40 wt% to 60 wt%, and found that the well-ordered layered structure was produced with the doctor-blading process. In addition, electrical conductivity was increased from 6670 S/m to 16,600 S/m [10]. Chen et al. [11] fabricated a highly ordered polyester/graphite flake composite by orienting the graphite flakes within a polymer matrix. They reported electrical property according to the orientation of the graphite flakes that are embedded in the polymer matrix. It was confirmed that the graphite flake-polymer composite had a better percolation path in the graphite flake alignment direction than that in the direction perpendicular to alignment, and electrical conductivity in the direction perpendicular to graphite alignment is five to six orders of magnitude higher than that parallel to the graphite alignment [11]. Cataldi et al. [14] have presented a flexible conductive cotton fabric nanocomposite that is produced with 30 wt% graphene nanoplatelets and thermoplastic polyurethane (TPU). They reported that the sheet resistance of cotton nanocomposite fabric was indicated under 10 Ω/sq, and they could withstand several cycles of weight-pressed severe folding-unfolding events and laundry at the same time.

Carbon-based nanomaterials are known to exhibit electric heating properties in accordance with Joule heating. Related studies [15,16,17,18,19,20,21,22,23,24] regarding electric heating elements are also steadily progressing. Generally, the heat generating mechanism of graphene-based electrical heater is in accordance with to Joule heating, a phenomenon that occurs when electrical current is passed through a material with an electrical resistance. The resistance that is inherent to a material leads to a conversion of electrical energy to thermal energy. This is caused by the collision of moving electrons with atoms that are main constituents of the material. Previous studies have reported that increasing the graphene contents in graphene/polymer composite will lead to an increase of electrical heating properties due to the increases of conductive network that form the continuous graphene layers [17,18,19,20]. An et al. [19] have reported the electrical heating performance of graphene/polymer composite film. The range of graphene content was from 0.0 wt% to 10.0 wt% and applied voltage range was from 0 V to 100 V. The results of electric heating behavior confirmed that the temperature of the composite film rapidly increased with increasing graphene contents and applied voltages. Thus, the surface temperature of 10 wt% graphene/polymer composite was indicated as 120 °C at an applied voltage of 30 V. In our previous study [22], using horseshoe pattern (HP), an electrical heating textile was prepared with 16 wt% graphene/graphene nanoplatelet/poly(vinylidene fluoride-*co*-hexafluoropropylene) (PVDF-HFP) composite that was coated on flame retardant cotton fabric. The width of HP was 2.5 mm and lengths of HP were 100 mm, 75 mm, and 50 mm, respectively. The sheet resistance of HP-pattern coated fabric decreased with decreasing the sample length and coated area from 73 kΩ/cm^2^ to 23 kΩ/cm^2^. The electrical heating property of HP showed an appearance of locally excess heat at the curved-shape of HP area. For surface temperature of HP samples with sample lengths of 100 mm, 75 mm, and 50 mm were 6.0 ± 2.4 °C, 6.8 ± 4.5 °C, and 3.5 ± 1.7 °C, respectively. It has been confirmed that heating performance is improved due to the collision of electrons in the curved region with decreasing sample size. The highest temperature also showed an increase of heating performance. Shin et al. [23] have reported a line-type patch film heater (EGnP-baed line heater) that was prepared with expanded graphite nanoplatelet. Temperatures of EGnP-based line heater at applied voltage of 4 V and 6 V were indicated at about 40 °C and 75 °C, respectively. With increasing voltage being applied to EGnP-based line heater, the temperature also increases [23].

Thus, in this study, we fabricated a horseshoe-pattern type graphene nanoplatelet/PVDF-HFP composite coated electrical heating textiles that exhibited temperature of a 50 °C at 5 V. This is a harmless electrical heating condition to the human body using a high content of graphene nanoplatelet. It can be applied to heating gloves and clothing in winter or under extreme conditions. The samples were coated on flame retardant cotton fabric with a horseshoe pattern by using the graphene nanoplatelet/PVDF-HFP composite. We tried to apply silver coated conductive yarn sewn in the graphene nanoplatelet/PVDF-HFP coated fabric as textile in order to improve their flexibility and wearability. The electrical and electrical heating properties of these samples were then analyzed.

## 2. Materials and Methods

### 2.1. Materials

Graphene nanoplatelets (GNP-UC, Carbon Nano Technology Co., Ltd., Pohang, Korea) and PVDF-HFP chips (SOLEF 21508, Solvay Co., Ltd., Brussels, Belgium) were used in this study. First-grade acetone (Junsei Chemical Co., Ltd., Tokyo, Japan) was used as the solvent. Flame retardant cotton fabric (Mirae Advanced Material Co., Ltd., Daejeon, Korea) with twill structure at thickness of 0.65 mm and weight per unit area of 0.042 g/cm^2^ was used as substrate material. Silver coated conductive yarn of 140 D (Soitex, Goyang, Korea) was used as an electrode. Commercial cotton yarn of 280 D was used as lower yarn for sewing. These samples were sewn with an industrial sewing machine (LS2-B736, Unicorn, Busan, Korea).

### 2.2. Preparation of Graphene Nanoplatelet/PVDF-HFP Composite Solutions

The high contents of graphene nanoplatelet/PVDF-HFP composite solution with various contents of graphene nanoplatelet were prepared for use in this study. To obtain graphene nanoplatelet/PVDF-HFP composite solution, 15 wt% of PVDF-HFP solution was prepared by placing PVDF-HFP chips in 1st grade acetone. Different contents (32 wt%, 40 wt%, 48 wt%, 56 wt%, and 64 wt%) of graphene nanoplatelets were then added into the as-prepared PVDF-HFP solution. These prepared graphene nanoplatelet/PVDF-HFP composite solution were stirred for more than one week with a digitally controlled hotplate stirrer (MSH-20D, Daihan scientific, Wonju, Korea) at room temperature to prepare the homogeneously dispersed solutions.

### 2.3. Preparation of Graphene Nanoplatelet/PVDF-HFP Composite Coated on the Fabric with Horseshoe Pattern

**Figure 1:**
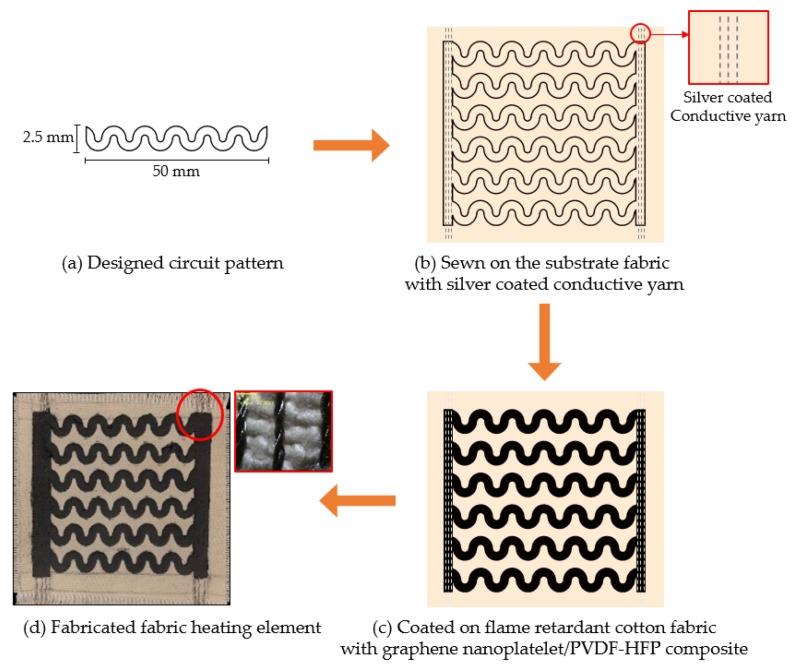
Preparation process of fabric heating element.

Figure 1 presented the scheme of a process of sample preparation of this study. Fabric heating elements with horseshoe-type (below HP) circuit pattern were prepared, as described previously [22]. The width and length of the HP were fixed at 2.5 mm and 50 mm, respectively. Untreated flame-retardant cotton fabric was prepared in the warp direction with a size of 60 mm × 60 mm to fabricate the fabric heating elements. Silver coated conductive yarn at upper position and commercial cotton yarn at lower position were sewn (the three lines) within 5 mm at both edges of samples. Six HP were then coated on the flame-retardant cotton fabric with a knife edge method using graphene nanoplatelet/PVDF-HFP composite. All of the prepared samples were subjected to a hot-press process at 140 °C for 3 min. at 3.5 MPa. Table 1 shows the sample code.

### 2.4. Characterization

#### 2.4.1. Morphology

The samples were measured with a fabric image analysis system (Nex measure Pro5 NTZ-6000, Bestec Vision Co. Ltd., Gunpo, Korea) at ×6.5 magnification and a field emission scanning electron microscope (FE-SEM, Inspect F50, FEI company, Hillsboro, OR, USA) at ×300, ×1000, ×8000 and ×20,000 magnifications to investigate the morphologies of coated cotton fabrics. The acceleration voltage was applied at 5 kV to measure the FE-SEM. Samples were visualized after a platinum coating process for 60 s.

#### 2.4.2. X-ray Diffraction

X-ray diffraction (XRD) spectra were obtained with X-ray diffractometer (Ultima IV, Rigaku Co. Ltd., The Woodlands, TX, USA) using Ni-filtered CuKα radiation and measurement range from 5° to 65° to confirm the crystal structure of graphene/PVDF-HFP coated samples with high content of graphene at various graphene loadings.

#### 2.4.3. Thermal Stability

The thermal stabilities of the samples were determined with thermogravimetric analysis (TGA, TA Instrument, New Castle, DE, USA) and differential scanning calorimetry (DSC 8500, PerkinElmer, Waltham, MA, USA). TGA was performed in oxygen at a heating rate of 20 °C/min. The measurement temperature ranged from 30 °C to 700 °C. DSC was conducted under nitrogen atmosphere with a starting temperature of 30 °C, which was increased up to 350 °C at a heating rate of 20 °C/min.

#### 2.4.4. Sheet Resistance

Sheet resistance was measured with a multimeter (ST850A, Saehan Tester. Co. Ltd., Busan, Korea) based on the AATCC-76 method to determine the electrical properties. Two parallel conductive prove were placed in contact with both edges of the samples. The sheet resistance (*R_s_*) is expressed in ohms per square. It was calculated according to Equation (1):

*R_s_*(Ω/sq) = (*W*/*D*) × *R* (1)

where *R* is resistance measured by the multimeter, *W* is the width of the sample, and *D* is the distance between the two electrodes.

#### 2.4.5. Electrical Heating Performance

Electrical heating performance was determined based on surface temperature with various applied voltages while using a DC power supply (CPS-2450B, CHUNPA EMT Co. Ltd, Bucheon, Korea). Both edges of samples consisting of silver coated conductive yarn were connected to the alligator clips. For application in clothing, this study applied low voltage from 0 V to 6 V with 1 V (DC) intervals for 3 min. A thermal imaging camera (FLIR i5, FLIR Systems INC., Wilsonville, OR, USA) was used to measure the surface temperature after applying different voltages. The current value was measured when the voltage was applied to the sample. Three samples were measured and the mean value was calculated.

**Figure 2:**
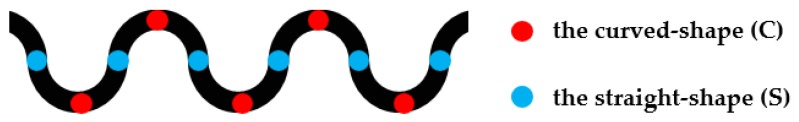
The position of the measured area of curved-shape (C) and straight-shape (S).

Time-dependent temperature changes of samples were measured with a temperature data logger (TR-71wf, T&D corp., Matsumoto, Japan) and a temperature sensor (TR-0206, T&D corp., Matsumoto, Japan) to confirm the part of local area. Figure 2 displays the position of the measured area for curved-shape and straight-shape. The samples were applied a voltage at 5 V for 30 min. The temperature values were then measured until the temperature reached an equilibrium state.

## 3. Results and Discussion

### 3.1. Morphology of GR/Cotton by Various High-Content of Graphene Nanoplatelets

Table 2 displays the morphology of GR/cotton coated with various high-content of graphene nanoplatelets at different magnifications. At low magnification, the surface of uncoated cotton presented a smooth surface and their surface confirmed that it was formed with cotton fibers. After coating with graphene nanoplatelet/PVDF-HFP composite, the surface of 32–64 GR/cotton formed a laminar layer, which indicated that graphene nanoplatelet/PVDF-HFP composites were successfully coated onto fabrics. The morphology images at higher magnification showed a coated layer on the cotton fabric in more detail. It was confirmed that the number of layers stacked with two-dimensional sheet type of graphene nanoplatelet particles was increased and the area of polymer used as binder was decreased with increasing graphene content. Hu et al. [10] have prepared a high-loading composite film containing 40–60 wt% graphene in graphene/PVDF composite film and reported that all of the films have layered structure. In addition, sheet-like layers of graphene nanoplatelets are parallel to film surface. They interpenetrate into adjacent layers. They reported that the oriented arrangement is attributed to the shear force that is imposed by doctor blade and further improved during subsequent drying [10]. Additionally, after hot pressing process, it is confirmed that more adjacent layers of graphene nanoplatelet/PVDF-HFP composite are indicated, and composite is impregnated in the samples [14]. In our previous study [24], we have confirmed that graphene/polymer coated sample with a hot pressing process have packed the voids with graphene dispersion. Thus, with increasing the high-content of graphene nanoplatelets in graphene nanoplatelet/PVDF-HFP composite, more overlapped graphene nanoplatelets formed an adjacent and continuous layer on the substrate fabric surface with conductive networks. 

### 3.2. XRD Analysis of GR/Cotton by Various High-Content of Graphene Nanoplatelets

**Figure 3:**
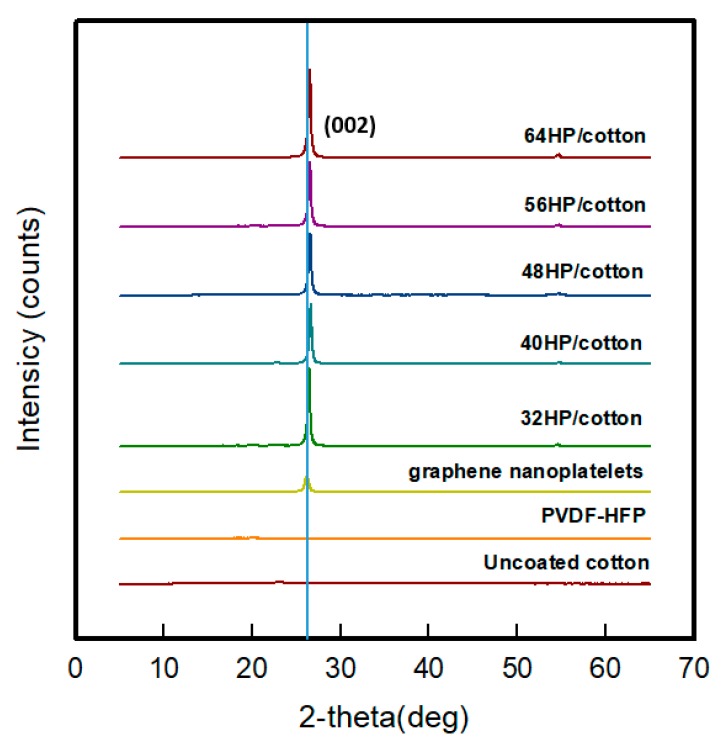
X-ray diffraction (XRD) pattern of GR/cotton coated with various high-content of graphene nanoplatelets.

Figure 3 shows the result of XRD analysis of GR/cotton coated with various high-content of graphene nanoplatelets. Generally, graphene nanoplatelets has a layer-by-layer structure. Thus, the XRD pattern of graphene has sharp peaks of (002) plane at 26.0° [6]. The characteristic peaks of α-phase of pure PVDF are at 2θ = 17.8°, 18.4°, 19.9°, 26.5°, and 38.9° allocated to (100), (020), (110), (021), and (002) planes, respectively [12]. Additionally, cotton fiber cellulose has four characteristic peaks that are located at 2θ = 14.7°, 16.6°, 27.7°, and 34.4°, indicated by (101), (10ī), (002), and (040) crystal reflections [25].

As shown in Figure 2, the highest diffraction peaks of the flame-retardant cotton fabric is observed at 2θ = 22.8°. The highest peak of graphene nanoplatelets and PVDF-HFP were indicated at 2θ = 26.1° of the (002) plane and 2θ = 19.9° of the (110) plane. In the case of GR/cotton, the dominant peak was found at 2θ = 26.6°, which was confirmed as a graphene nanoplatelets peak. The peaks of cotton fabric and PVDF-HFP almost disappeared. This confirmed that the high-content of graphene nanoplatelets/PVDF-HFP composite coated on the uncoated cotton entirely. Additionally, intensities of peaks of (002) plane of GR/cotton were orderly increased by increasing the high-content of graphene nanoplatelets from 32 GR/cotton to 64 GR/cotton. Chen et al. [11] reported the highly ordered alignment of graphite flakes that were embedded in the polymer. One was a graphite flake plane facing (perpendicular to) the X-ray scanning direction during testing, while the other was parallel to the X-ray scanning direction. In that report, the significant peak at 2θ = 26.4° attributed to (002) crystalline lamellae of graphite was indicated as perpendicular to the X-ray scanning direction. This result indicated that, as the graphene nanoplatelets content within the graphene nanoplatelets/PVDF-HFP composite increased, the diffraction peak of (002) plane facing the X-ray scanning direction increased, since more overlapped graphene particles formed a stacked and continuous layer.

### 3.3. Thermal Stability of GR/Cotton by Various High-Content of Graphene Nanoplatelets

#### 3.3.1. Thermogravimetric Analysis (TGA)

**Figure 4:**
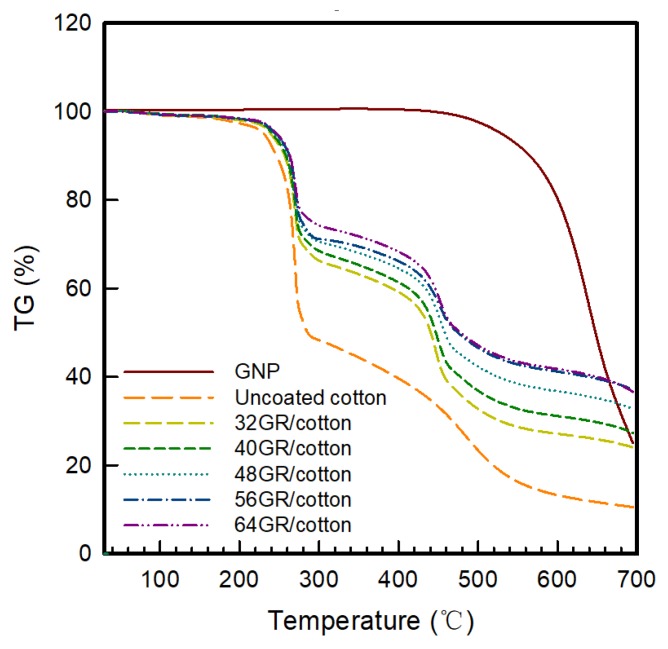
Thermogravimetric analysis (TGA) curve of GR/cotton coated with various high-content of graphene nanoplatelets.

The thermal degradation process of GR/cotton with various high-content of graphene nanoplatelets was investigated by thermo-gravimetric analysis in oxygen. The results are shown in Figure 4 and Table 3. Yang et al. [13] have evaluated the thermal properties of polyvinylidene fluoride (PVDF)/graphene nanoplatelets (GNP) composite. GNP was added with 0 to 8 wt% into PVDF solution. They reported that PVDF began its degradation at 488.7 °C. With increasing the GNP contents from 0 wt% to 8 wt%, the degradation temperature was indicated from 488.7 °C to 491.2 °C. The first stage started at around 225 °C, corresponding to the decomposition temperature of cotton fabric, while the second stage was started at 430 °C, corresponding to the decomposition temperature of PVDF-HFP. The TGA curve confirmed that graphene nanoplatelets/PVDF-HFP composite coating improved the thermal stability of pristine cotton fabric. When compared with uncoated cotton, GR/cotton showed improvement in the onset temperature and temperature of the maximum degradation. The uncoated cotton started to degrade at about 227.4 °C. Its weight loss rate reached the 1st transition temperature (1st T_trans_) and 2nd maximum temperature (2nd T_max_) at 287.1 °C and 444.6 °C. The residue was 49.0% at the 1st T_trans,_ 35.1% at the 2nd T_max_, and 11.0% at 650 °C, respectively. The onset temperature of GR/cotton with various high-content of graphene nanoplatelets ranging from 32 wt% to 64 wt% ranged from 230.1 °C to 234.1 °C, and reaching the 1st T_trans_ of 277.3 °C to 293.2 °C and 2nd T_max_ of 457.1 °C to 468.6 °C. The residue rate at reached 1st T_trans_, 2nd T_max_, and 650 °C for 32 GR/cotton to 64 GR/cotton ranged from 67.7% to 74.4%, 39.4% to 51.3%, and 25.3% to 39.4%, respectively. Yang et al. [13] have also reported that the char residues increased with increasing filler content. It was confirmed that the decrease of char residue with increasing temperature suggested that the slow decomposition of the carbon skeleton occurred after 500 °C, and the resulting free radicals were subsequently transferred to the PVDF chain, which slightly reduced the degradation rate of the composites. Thus, the thermal stability was improved.

#### 3.3.2. Differential Scanning Calorimetry (DSC)

**Figure 5:**
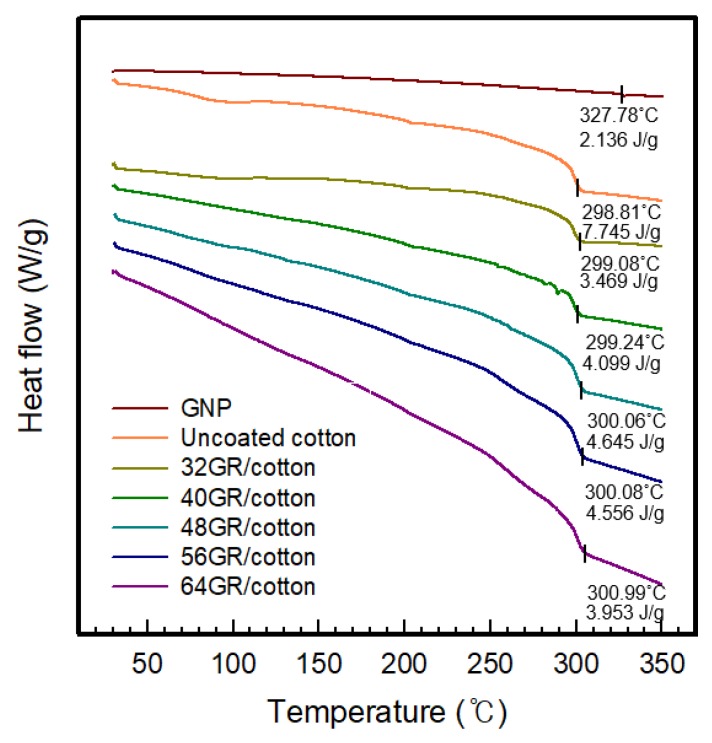
Differential scanning calorimetry (DSC) curve of GR/cotton coated with various high-content of graphene nanoplatelets.

Figure 5 presents the results of the DSC curves performed for uncoated cotton and GR/cotton with various high-content of graphene nanoplatelets. As shown in Figure 5, the uncoated cotton that is used in this study is a flame-retardant cotton with a decomposition temperature at 298 °C. The decomposition temperature tended to increase from 298.8 °C to 301.0 °C when the graphene content was increased from 32 GR/cotton to 64 GR/cotton. The results showed that the thermal stability of GR/cotton as improved with increasing the high-content of graphene nanoplatelets. Therefore, 64 HP/cotton possessed the best thermal stability with the highest decomposition temperature.

### 3.4. Sheet Resistance of GR/Cotton by Various High-Content of Graphene Nanoplatelets

**Figure 6:**
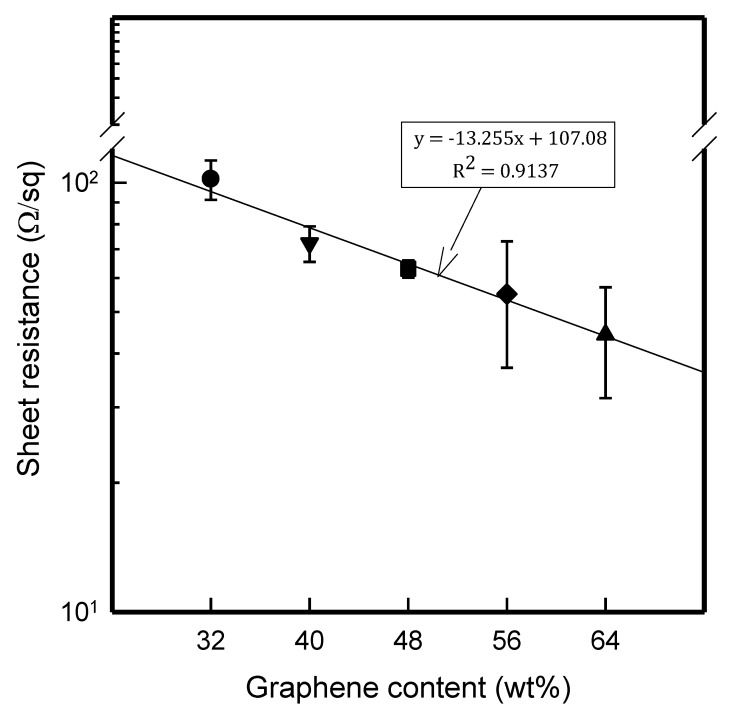
Sheet resistance of GR/cotton coated with various high-content of graphene nanoplatelets.

The sheet resistance of GR/cotton coated with various high-content of graphene nanoplatelets was measured to determine the electrical properties. Figure 6 displays the results. As shown in Figure 6, the sheet resistance of GR/cotton samples tended to linearly decrease with increasing graphene nanoplatelets contents with *R*^2^ of 0.9137. The sheet resistance of 32 GR/cotton, 40 GR/cotton, 48 GR/cotton, 56 GR/cotton, and 64 GR/cotton were 1.0 × 10^2^ ± 1.0 × 10^1^ Ω/sq, 7.2 × 10^1^ ± 6.8 Ω/sq, 6.3 × 10^1^ ± 2.9 × 10^1^ Ω/sq, 5.5 × 10^1^ ± 1.7 × 10^1^ Ω/sq, and 4.4 × 10^1^ ± 1.3 × 10^1^ Ω/sq, respectively. Previous studies on electrical properties of films or coated fabrics that were made of graphene/polymer composites have reported that the sheet resistance decreases with increasing graphene contents in the composite [8,9,10,11,12,13,14]. It has been reported that the increase of graphene nanoplatelets in the graphene nanoplatelets/polymer composite can lead to the formation of a conductive network consisting of conductive nanoparticles in the polymer matrix. This result demonstrates that, when the number of physical contacts between graphene nanoplatelets content increases and the more electric charges can flow through the GR/cotton.

The electrical properties of GR/cotton coated with various high contents of graphene nanoplatelets were confirmed to be related to the crystal structure. As mentioned above, the electrical conductivity in the direction perpendicular of the graphite/polymer composite to the graphite alignment have been reported to be five to six orders of magnitude higher than that parallel to the graphite alignment, since the increase in the electric anisotropy can be attributed to the flake-like shape of graphite flakes and their higher degree of alignment [11]. The results of the present study confirmed that, when graphene content within the graphene/PVDF-HFP composite increased, the diffraction peak of (002) plane facing the X-ray scanning direction also increased. Therefore, the high-content of graphene nanoplatelets developed the (002) plane and the surface resistivity showed that the decrease graphene nanoplatelets content was increased. The sheet resistance value of 64 GR/cotton was the lowest.

### 3.5. Electrical Heating Properties of GR/Cotton by Various High-Content of Graphene Nanoplatelets

#### 3.5.1. Electrical Heating Property at Various Applied Voltages

**Figure 7:**
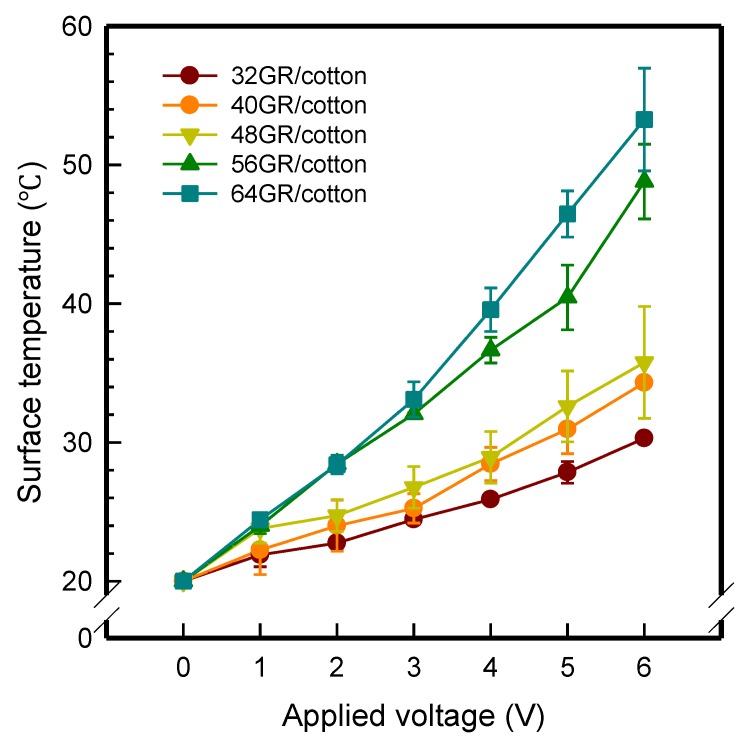
Variation of surface temperature of GR/cotton coated with various high-content of graphene nanoplatelets.

To investigate the electrical heating property of GR/cotton coated with various high-content of graphene nanoplatelets, surface temperatures of samples were confirmed while using IR thermal images. The surface temperature of each sample was measured at various applied voltages from 1 V to 6 V with intervals of 1 V for 3 min. Figure 7 shows the variation of surface temperature of samples by different applied voltages and IR images of samples at 5 V. The heat generated across the graphene nanoplatelets/PVDF-HFP coated layer is a result of the Joule heating phenomenon [14]. Joule heating is a phenomenon that occurs when an electrical current is passed through a material with an electrical resistance. The resistance that is inherent to the material leads to a conversion of electrical energy to thermal energy. This is caused by the collision of moving electrons with atoms that are constituents of the main material.

To evaluate electrical heating properties of graphene/polymer or carbon-nano material/ polymer composite based heater, copper materials, such as copper plate, copper wire, and copper tape, has been mainly used as electrode [22,23,24]. These conductive materials were used to lower the contact resistance between the power source and graphene or carbon-nano material-based heater and maintain the controlled output voltage. In this study, to improve the flexibility, wearability, and applicability of electrical heating textile, we used silver coated conductive yarn as the electrode for the GR/cotton samples. To confirm the distribution of heating area of the GR/cotton samples, the IR images of surface temperature of GR/cotton with various high-content of graphene nanoplatelets at 5 V are shown in Table 4. Heat generation was observed throughout GR/cotton samples. The surface temperature and current of GR/cotton were increased with increasing graphene nanoplatelet content. When the graphene nanoplatelet content was increased, graphene nanoplatelets within the polymer were increased. They could form more conductive paths. Thus, the electrical heating performance was improved by increasing the interaction between the graphene nanoplatelets. The results confirmed that 64 GR/cotton had good electrical heating property.

As shown in Figure 7, surface temperatures of GR/cotton with various high-content of graphene nanoplatelets were increased when the applied voltage increased according to Joule’s law [15,16,17,18,19,20,21,22,23,24]. The surface temperature of GR/cotton was also improved as the high-content of graphene nanoplatelets increased from 32 GR/cotton to 64 GR/cotton. Shin et al. [23] have reported a line-type patch film heater (EGnP-baed line heater) that was prepared with expanded graphite nanoplatelet. The temperature of EGnP-based line heater at applied voltage of 5 V and 6 V were indicated as about 40 °C and 75 °C, respectively. With increasing voltage being applied to EGnP-based line heater, the temperature of EGnP-based line heater also increases. In this study, when voltage was applied at 5 V, the surface temperatures of 32 GR/cotton, 40 GR/cotton, and 48 GR/cotton were less than around 30 °C, whereas 56 GR/cotton and 64 GR/cotton were about 42 °C and 48 °C, respectively. This result confirmed that the 64 GR/cotton could be indicated at about 50 °C when a low voltage between at 5 V and 6 V was applied.

#### 3.5.2. Time-Dependent Temperature Changes

**Figure 8:**
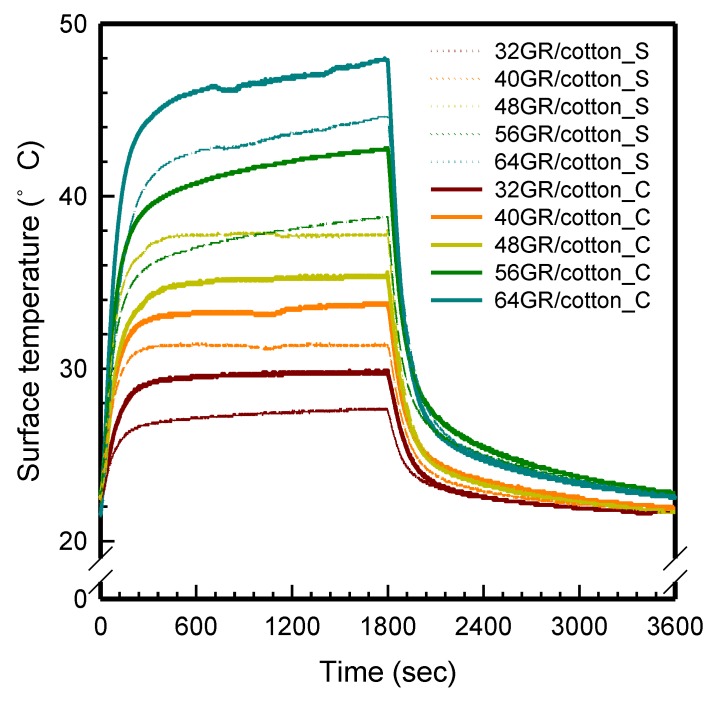
Time-dependent temperature of local area of GR/cotton coated with various high-content of graphene nanoplatelets at 5 V (Dot line: curved-shape (C) and Solid line: straight-shape (S)).

Figure 8 displays the time-dependent temperature changes of GR/cotton that was coated with various high-content of graphene nanoplatelets at 5 V. In our previous study [22], the horseshoe pattern has two parts of heating area due to the collision of electrons in the curved-shape region. In addition, electrical heating property tended to increase as the size of sample decreased from 100 mm × 100 mm to 50 mm × 50 mm. As shown in Figure 8, the temperature curves are divided into two areas. One is a curved-shape area and the another is a straight-shape area, as shown in Figure 2. Differences between curved-shape and straight-shape of 32 GR/cotton, 40 GR/cotton, and 48 GR/cotton were within 3.0 °C. However, 56 GR/cotton and 64 GR/cotton presented temperature differences of 4.9 ± 1.1 °C and 5.7 ± 1.6 °C, respectively. This indicates that the curved-shape region of the horseshoe pattern has more collision of electrons than the straight-shape region. When the electrical heating property is increased, the collision between graphene nanoplatelets is also increased when the current is supplied. This is because the conductive network is formed due to excessive graphene. Thus, electrical heating temperature gradually increases with increasing graphene nanoplatelets content [22].

As shown in Figure 8, the GR/cotton samples could reach a steady-state temperature within 10 min. and maintain it for 20 min. When the applying voltage was turned off, the temperature was decreased to room temperature within 5 min. In case of 56/cotton and 64/cotton, the maximum surface temperature was slightly increased during 30 min. This due to an increase electric charge flows in the graphene nanoplatelets/PVDF-HFP coated area, owing to the electric heating effect. Generally, the quantity of heat (*Q*) yielded from the applied electrical energy is calculated according to Equation (2):

*Q* = *I*^2^*RT* (2)

where *I* is current, *R* is resistance, and *T* is period of the time.

This result indicates that 56 GR/cotton and 64 GR/cotton were more affected than 32 GR/cotton, 40 GR/cotton, and 48 GR/cotton by the current value according to period of time. Therefore, the long-lasting electrical heating behavior was confirmed in the GR/cotton with various high-content of graphene nanoplatelets, with 64 GR/cotton presenting the best electrical heating performance and behavior.

## 4. Conclusions

In summary, this study prepared horseshoe pattern type of graphene nanoplatelet/PVDF-HFP composite with high-content of graphene nanoplatelet that was coated on flame retardant cotton fabric (GR/cotton) as a fabric heating element GR/cotton was coated with graphene nanoplatelet/PVDF-HFP composite with high-content of graphene nanoplatelet ranging from 32 wt% to 64 wt%, with intervals of 8 wt%. To evaluate the electrical heating properties of fabric heating elements, copper materials, such as copper plate, copper wire, and copper tape, has been mainly used as electrode. However, those are difficult to apply to wearable textile, because they have brittle, corrode, and tarnish over time. In our study, to improve its flexibility and applicability as a wearable textile, silver coated conductive yarn was used as the electrode of GR/cotton. With increasing the high-content of graphene nanoplatelet in GR/cotton, stacked and overlapped graphene nanoplatelet layers were also increased. It improved the crystallinity of graphene nanoplatelet peak 2θ = 26.6° and thermal stability. The sheet resistance of the GR/cotton sample tended to linearly decrease with an increasing high-content of graphene nanoplatelet. It was decreased by half from 32 GR/cotton to 64 GR/cotton. Furthermore, the electrical heating properties of GR/cotton were improved with increasing the high-content of graphene nanoplatelet. When 5 V was applied to 64 GR/cotton, the surface temperature was indicated at about 48 °C. Additionally, samples could reach steady-state temperature within 10 min. and maintained the temperature for 30 min. Therefore, the horseshoe-pattern type of graphene nanoplatelet/PVDF-HFP composite with high-content of graphene nanoplatelet coated on flame-retardant cotton fabric (GR/cotton) is expected to have applications as wearable fabric heating elements, such as gloves, shoes, jacket, and so on for use in winter and extreme conditions.

## Figures and Tables

**Table 1:** Sample codes of fabricated samples coated with graphene nanoplatelet/poly(vinylidene fluoride-*co*-hexafluoropropylene) (PVDF-HFP) composite with various high-content of graphene nanoplatelet.

Graphene Nanoplatelet Contents (wt%)	Sample Code
0	Uncoated cotton
32	32 GR/cotton
40	40 GR/cotton
48	48 GR/cotton
56	56 GR/cotton
64	64 GR/cotton

**Table 2:** Morphology of GR/cotton coated with various high-content of graphene nanoplatelets.

Sample Code	Magnification				
	6.5	300	1000	8000	20,000
Uncoated cotton	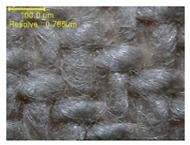	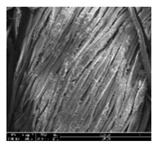	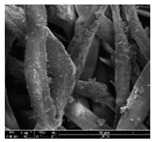	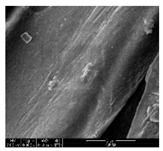	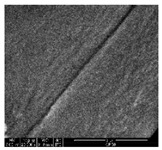
32 GR/cotton	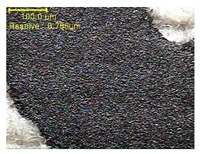	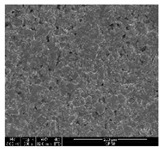	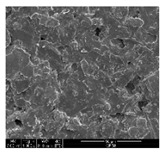	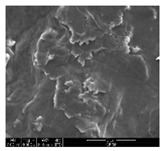	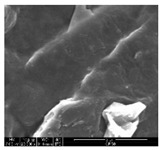
40 GR/cotton	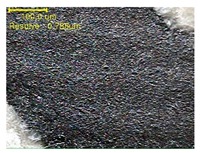	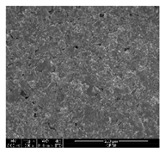	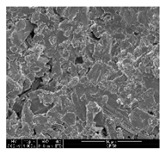	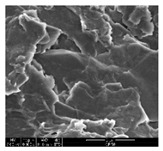	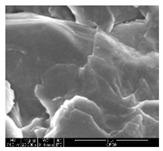
48 GR/cotton	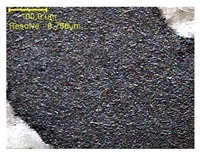	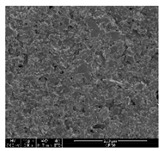	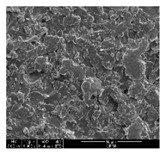	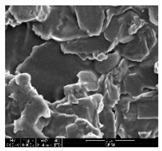	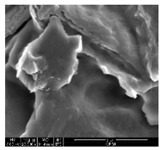
56 GR/cotton	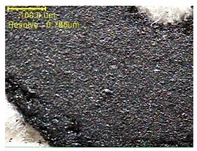	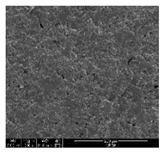	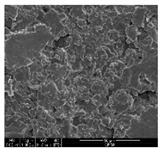	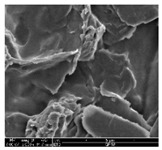	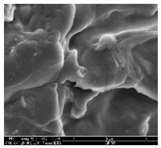
64 GR/cotton	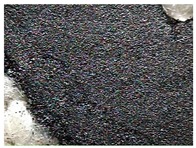	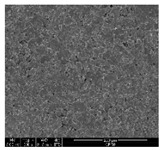	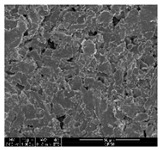	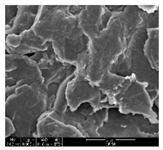	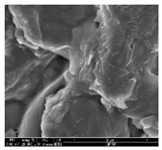

**Table 3:** Thermal stability properties of GR/cotton coated with various high-content of graphene nanoplatelets.

Sample Code	TGA					
	* T_onset_ (°C)	1st		2nd		Residue at 650 °C (%)
		** T_trans_ (°C)	Residue at T_trans_ (%)	*** T_max_ (°C)	Residue at T_max_ (%)	
Graphene nanoplatelet	463.7	-		-		49.2
Uncoated cotton	227.4	287.1	49.0	444.6	35.1	11.0
32 GR/cotton	230.1	277.3	67.7	457.1	39.4	25.3
40 GR/cotton	230.5	280.8	70.4	461.6	42.4	29.4
48 GR/cotton	231.7	287.5	71.4	464.3	46.7	34.8
56 GR/cotton	232.8	291.8	71.6	467.4	49.8	38.7
64 GR/cotton	234.1	293.2	74.4	468.6	51.3	39.4

* T_onset_: Onset temperature; ** T_trans_: Transition temperature; *** T_max_: Maximum temperature.

**Table 4:** IR thermal images and current of GR/cotton coated with various high-content of graphene nanoplatelets at 5 V.

Applied at 5 V	Sample Code				
	32 GR/Cotton	40 GR/Cotton	48 GR/Cotton	56 GR/Cotton	64 GR/Cotton
IR image	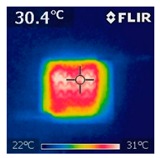	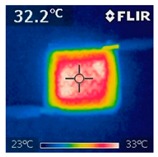	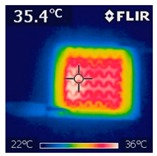	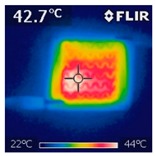	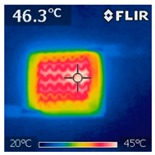
Current (A)	0.07 ± 0.01	0.09 ± 0.00	0.12 ± 0.02	0.15 ± 0.01	0.20 ± 0.04
